# Treatment of mariculture wastewater by indigenous cyanobacteria-bacteria consortium in a photobioreactor

**DOI:** 10.1128/spectrum.02807-25

**Published:** 2026-03-18

**Authors:** Liyun Cai

**Affiliations:** 1 Xiamen University Tan Kah Kee College 523205, Zhangzhou, China; Mikrobiologický ústav AV ČR, v. v. i., Třeboň, Czechia

**Keywords:** indigenous cyanobacteria-bacteria consortium, treatment performance, mariculture wastewater, photobioreactor, *Ulva *sp. UNA00071828

## Abstract

The bioexploration of indigenous bacterial and algal species presents a promising approach for local wastewater treatment. In this study, an indigenous cyanobacteria-bacteria consortium was collected from rock surfaces at the discharge outlet of mariculture wastewater in Zhangzhou City, China, and subsequently inoculated into a photobioreactor (PBR) for treating both simulated and actual mariculture wastewater. The effects of influent pollutant concentration, consortium inoculation mass, and hydraulic retention time on the treatment performance of the PBR were systematically investigated. Results indicated that the PBR inoculated with this indigenous bacteria-algae consortium exhibited strong adaptability in treating both simulated and actual mariculture wastewater across varying pollutant concentrations. Furthermore, the PBR achieved significant removal of total organic carbon, total nitrogen, nitrite nitrogen, ammonia nitrogen, and total phosphorus from actual shrimp mariculture wastewater within a short period (2–4 h), while considerably enhancing the dissolved oxygen level of the treated effluent. High-throughput sequencing revealed that the consortium was primarily composed of the bacterial phyla Pseudomonadota, Bacteroidota, and Cyanobacteriota, with the dominant species being green cyanobacteria *Ulva* sp. UNA00071828.

**IMPORTANCE:**

This study developed a sustainable approach for mariculture wastewater treatment by utilizing indigenous biofilm aggregates—comprising bacteria and algae—harvested directly from a local wastewater discharge outlet. Key operational parameters, including inoculum mass, influent pollutant concentration, and hydraulic retention time, were systematically evaluated to optimize treatment performance in a photobioreactor. Furthermore, shifts in microbial community composition and relative abundance were analyzed before and after operation. The results provide valuable insights for the design of efficient and low-cost biological treatment systems tailored to mariculture effluent.

## INTRODUCTION

In recent years, the aquaculture industry has experienced rapid global expansion (1), accompanied by a substantial increase in mariculture wastewater (MWW) discharge. MWW is primarily characterized by high concentrations of organic matter, nitrogen (N), and phosphorus (P) (2). Current treatment approaches include physical, chemical, and biological methods (3). Nevertheless, conventional physical processes, such as mechanical filtration and foam separation, often fall short of achieving advanced purification standards (4). Similarly, widely applied chemical treatments—including ultraviolet irradiation, ozonation, neutralization, and chemical precipitation—frequently lead to the formation of undesirable by-products. In contrast, biological treatment processes are more commonly adopted due to their relatively lower operational costs compared with physical or chemical methods. Techniques, such as activated sludge, biofiltration, and biofilm-based systems, have demonstrated effectiveness in removing organic matter, N, and P (5). However, these biological methods are not without drawbacks, as they may introduce secondary pollutants, such as residual sludge.

Wastewater treatment systems based on photosynthetic microorganisms have emerged as a sustainable alternative to conventional biological processes (6). The concept of employing cyanobacteria for effluent treatment was first introduced by Oswald and Gotaas (7). Over the past decade, the application of cyanobacteria and cyanobacterial biofilms in treating wastewater from diverse sources has gained considerable research interest and has shown promising efficacy in the removal of both organic and inorganic pollutants (8). The cyanobacterial biofilm matrix provides protection to microbial communities against external environmental stressors (9). This approach presents several advantages, including lower operational costs—due to the elimination of mechanical aeration through photosynthesis—reduced CO_₂_ emissions, and the avoidance of chemical fertilizers for microbial cultivation, as nutrients are sourced directly from the wastewater (10). Moreover, the biomass produced can be further valorized as a feedstock for biofuel production (10, 11).

A persistent biofilm, composed of a cyanobacteria-bacteria consortium (CBC), is consistently observed on the stone surfaces at MWW discharge outlets. This indigenous biofilm has naturally adapted to the MWW environment and may offer distinct advantages over artificially constructed bacterial-algal consortia for wastewater treatment applications. First, unlike free-floating microalgae, which often present challenges related to sedimentation and harvesting, naturally attached algal and microbial communities form stable biofilms on solid substrates, effectively overcoming separation difficulties. Second, this spontaneously assembled mixed biofilm harbors a diverse array of microorganisms already acclimated to the wastewater conditions, enabling efficient nutrient uptake and potentially lowering operational costs and environmental impacts.

Accordingly, this study aims to evaluate the potential of a photobioreactor (PBR) inoculated with this indigenous CBC for treating both simulated and actual MWW. The specific research objectives are (i) to investigate the effects of consortium inoculum qualities on the removal efficiencies of nitrite nitrogen (NO₂⁻-N), ammonia nitrogen (NH₄^+^-N), total nitrogen (TN), total phosphorus (TP), and total organic carbon (TOC) and (ii) to characterize the dynamic changes in the microbial composition and relative abundance within the consortium. The findings are expected to contribute to the development of an economically feasible and environmentally suitable process for MWW treatment.

## MATERIALS AND METHODS

### Simulated MWW

The simulated MWW was prepared by homogenizing seawater, commercial fish feed, and krill using a blender. Specifically, a measured quantity of fish feed was initially soaked in natural seawater collected from the Taiwan Strait. The soaked feed was then blended into a uniform paste. Subsequently, crushed krill and additional seawater were added to obtain a high-concentration simulated wastewater stock. This stock solution was stored at −20°C until further use.

### Actual MWW

The actual MWW used in this study was collected from a local *Penaeus monodon* aquaculture pond in the Longhai District, Zhangzhou City, China. After collection, the wastewater was allowed to settle for 2 h to remove suspended solids and was subsequently stored at 4°C until use. Prior to experimentation, the wastewater was analyzed for TOC, inorganic carbon (IC), TN, TP, NH₄^+^-N, NO₂⁻-N, dissolved oxygen (DO), and pH, following the procedures outlined in “Water quality analysis,” below. The measured parameters were as follows: TOC, 10.68 ± 0.54 mg/L; IC, 27.25 ± 1.36 mg/L; TN, 4.45 ± 0.13 mg/L; TP, 0.84 ± 0.03 mg/L; NH₄^+^-N, 1.72 ± 0.05 mg/L; NO₂⁻-N, 0.02 ± 0.01 mg/L; pH, 6.92–7.07; and DO, 3.96–4.29 mg/L.

### Indigenous CBC

**Fig 1:**
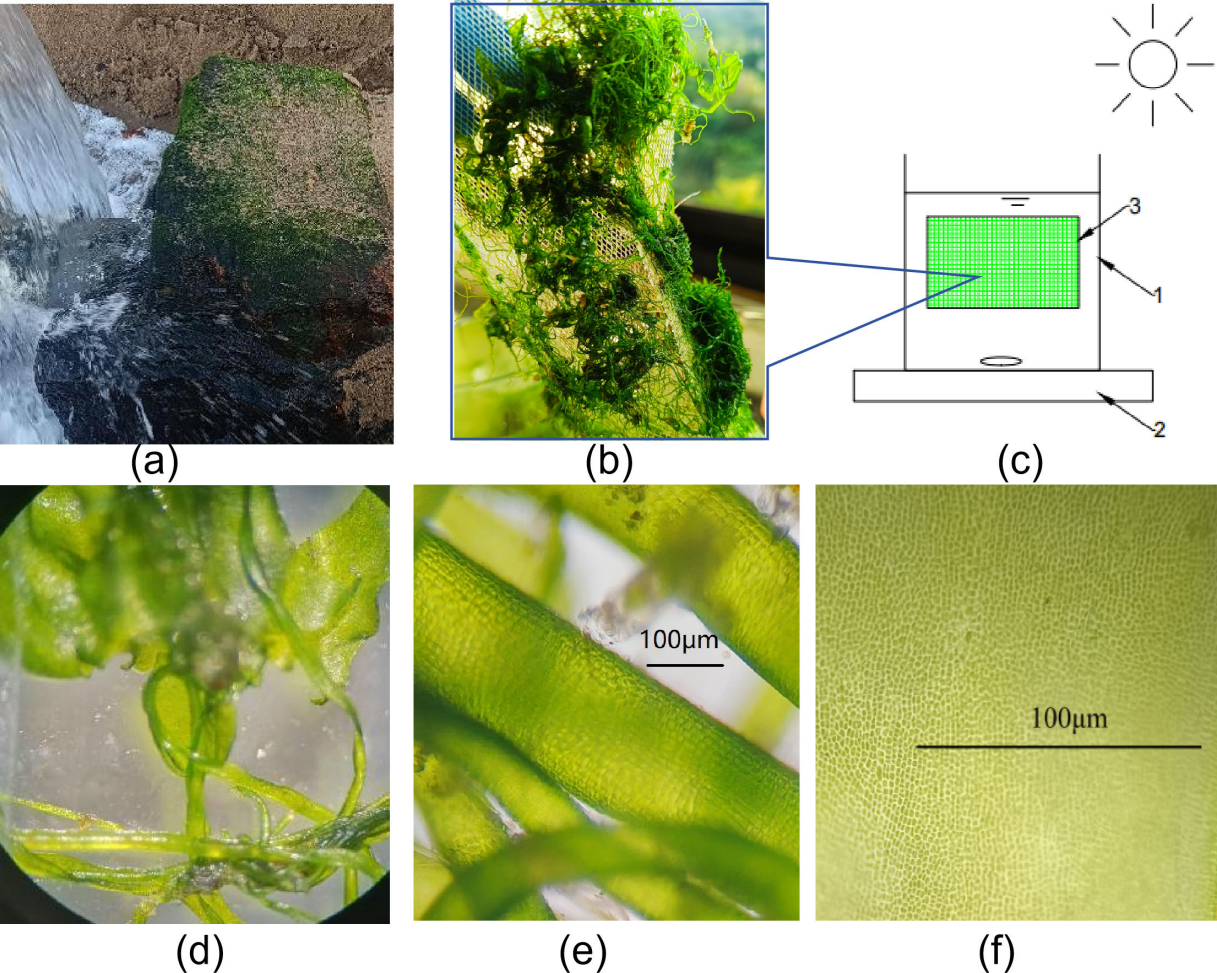
(**a**) Sampling areas for CBC; (**b**) CBC attached to the net in the PBR; (**c**) schematic diagram of PBR: 1—Glass material reactor body; 2—Magnetic stirrer; 3—Nylon net for CBC attachment; (**d–f**) macroalgae *Ulva* sp. UNA00071828.

The indigenous CBC used in this study was obtained from native biofilm growing on rock surfaces within the drainage channels of local mariculture facilities (24°16′N, 118°7′E) (Fig. 1a). Prior to inoculation, the CBC was rinsed with seawater to remove sediments and then adhered to a 50-mesh nylon net installed inside the PBR for subsequent operational experiments (Fig. 1b).

The consortium was dominated by green, filamentous, and readily adhesive cyanobacteria. Based on morphological examination and second-generation amplicon sequencing data, the main cyanobacteria was identified as *Ulva* sp. UNA00071828 (Fig. 1d through f), belonging to the phylum Cyanobacteriota, class Cyanobacteriia, and order Chloroplast.

### Experimental apparatus

The experiments were conducted in open PBRs (Fig. 1c). These PBRs were placed on a magnetic stirrer (HH-2J, China) to achieve continuous mixing of the wastewater and inoculum within the reactor through stirring at a speed of 280–300 rpm. During the experiment, the PBRs were positioned on a table next to an indoor window, where sunlight passing through the glass window served as the light source, providing a natural light-dark cycle. The sunlight intensity was measured using an illuminance meter (TES-1330A, Taishi). During the experiment, pH and temperature (ambient temperature was 25°C–32°C) in PBRs were not controlled, no additional air stirring was carried out, and no CO_2_ was introduced into the PBRs.

### Experiment design

#### Simulated MWW batch treatment experiment

In this study, batch experiments were conducted to evaluate the treatment performance of PBRs inoculated with the CBC on simulated MWW with varying influent concentrations. Treatment efficiency was assessed in terms of hydraulic retention time (HRT) and effluent quality.

Specifically, 10 g of wet CBC (prepared as described in “Indigenous CBC,” above) was inoculated into each PBR. Different volumes of simulated MWW (prepared according to “Simulated MWW,” above) were then added to achieve distinct initial pollutant concentrations, and the total liquid volume in each reactor was adjusted to 1 L using sterilized seawater. The PBRs were placed on magnetic stirrers and operated under natural sunlight, following the ambient light-dark cycle (as outlined in “Experimental apparatus,” above). Water quality parameters were measured at the start of the experiment and at predetermined time intervals. No medium exchange was performed during each batch run. After one batch (typically within 3 days), the treated wastewater was drained while retaining the attached CBC. The procedure was repeated by re-filling the PBRs with fresh simulated wastewater and sterilized seawater for subsequent batches.

#### Actual MWW batch treatment experiment

This study also evaluated the treatment effect of PBRs inoculated with different quantities of CBC on *Penaeus monodon* pond MWW. The treatment effect was evaluated based on HRT and effluent quality. Three groups of PBRs were inoculated with 5, 10, and 15 g of wet CBC (prepared as described in “Indigenous CBC,” above), respectively. Each PBR was then filled with 1 L of actual wastewater (see “Actual MWW,” above). All other experimental conditions followed the protocol outlined in “Experimental apparatus,” above. Water quality parameters were measured at the beginning of the experiment and at regular time intervals thereafter. Upon completion of each batch, the treated wastewater was replaced with fresh wastewater (Actual MWW) to conduct the subsequent treatment cycle.

### Water quality analysis

During operation, water samples were collected from the PBRs at predetermined intervals. After filtration through a 0.45-μm syringe filter, the samples were promptly analyzed for the relevant water quality indicators. TOC and total IC were quantified via high-temperature catalytic combustion using a TOC analyzer (TOC-L, Shimadzu, Japan). TN was determined by high-temperature catalytic oxidation coupled with chemiluminescence detection on the same instrument. TP was analyzed following persulfate digestion and the molybdate spectrophotometric method (12). NH₄^+^-N was measured using Nessler’s reagent spectrophotometry, and NO₂⁻-N was determined by the corresponding spectrophotometric method (12). DO was recorded with a DO meter (Leici, China), and pH was measured using a multiparameter meter (PHSJ-3F, Mettler-Toledo, China).

### Analysis of microbial species and abundance

#### DNA extraction and PCR amplification

After treating the actual MWW from shrimp pond for 9 days, total microbial genomic DNA was extracted from the indigenous CBC and CBC samples collected from the three PBRs using the E.Z.N.A. Soil DNA Kit (Omega Bio-tek, Norcross, GA, USA). The hypervariable V3–V4 region of the bacterial 16S rRNA gene was amplified with the primer pair 338F (5′-ACTCCTACGGGAGGCAGCAG-3′) and 806R (5′-GGACTACHVGGGT WTCTAAT-3′) in a T100 Thermal Cycler (Bio-Rad, USA). The PCR mixture contained 4 μL of 5× Fast Pfu buffer, 2 μL of 2.5 mM dNTPs, 0.8 μL of each primer (5 μM), 0.4 μL of Fast Pfu polymerase, 10 ng of template DNA, and ddH₂O to a final volume of 20 μL. The thermal cycling program consisted of initial denaturation at 95°C for 3 min; 27 cycles of denaturation at 95°C for 30 s, annealing at 55°C for 30 s, and extension at 72°C for 45 s; a final extension at 72°C for 10 min; and a hold at 4°C. PCR products were separated on a 2% agarose gel, purified with a PCR Clean-Up Kit (YuHua, Shanghai, China), and quantified using a Qubit 4.0 fluorometer (Thermo Fisher Scientific, USA).

#### Illumina sequencing

Purified amplicons were pooled in equimolar amounts and paired-end sequenced on an Illumina Nextseq2000 platform (Illumina, San Diego, USA) according to the standard protocols by Majorbio Bio-Pharm Technology Co. Ltd. (Shanghai, China).

#### Statistical analysis

Bioinformatic analysis of the microbiota was carried out using the Majorbio Cloud platform (https://cloud.majorbio.com).

### Growth analysis

Biomass productivity in the PBR was calculated by dividing the difference in dry weight at the end and start of the experiment.

Biomass productivity [*P_x_*, gDW/(d·L)] was calculated as follows:


$$P_{x}=\frac{DW_{f}-DW_{o}}{t_{f}-t_{o}}$$

where DW*_f_* (g/L) is the dry weight at the end of the experiment (time *t_f_*), whereas DW_*0*_ (g/L) is the dry weight at the start of the experiment (time *t*_*0*_).

## RESULTS AND DISCUSSION

### Morphology and microscopic images of the CBC

**Fig 2:**
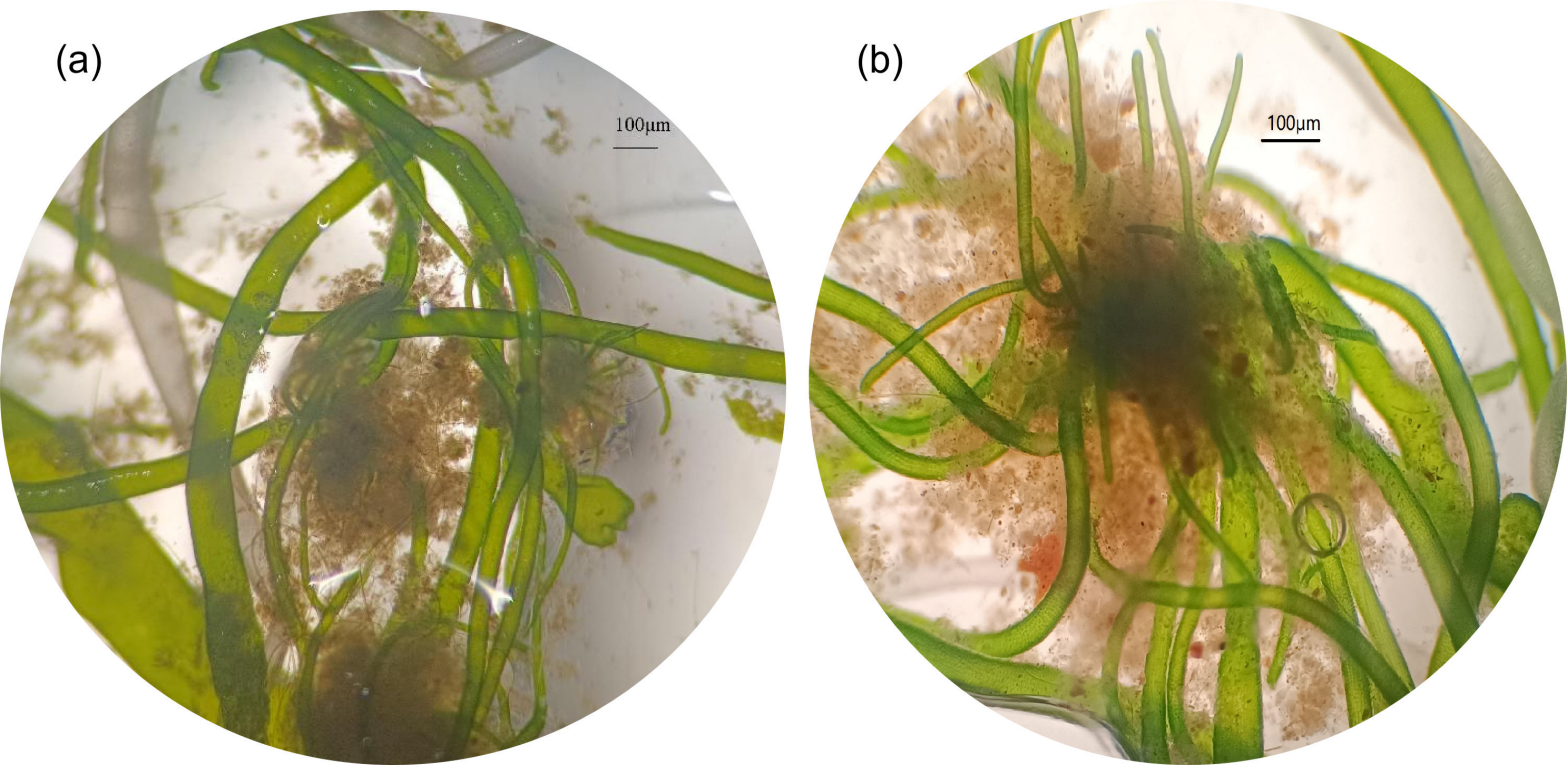
Morphology and microscopic images of the CBC in PBRs treating MWW. (**a**) The combined form of bacteria and algae; (**b**) bacterial and algal polymers.

After a period of MWW cultivation, microscopic examination revealed that the consortium was predominantly composed of green filamentous macroalgae, with bacterial communities visibly associated with the algal branches (Fig. 2a). Algal filaments were observed extending outward from bacterial aggregates, as shown in Fig. 2b.

### Treatment effect of PBR on simulated MWW of different pollutants concentrations

**Fig 3:**
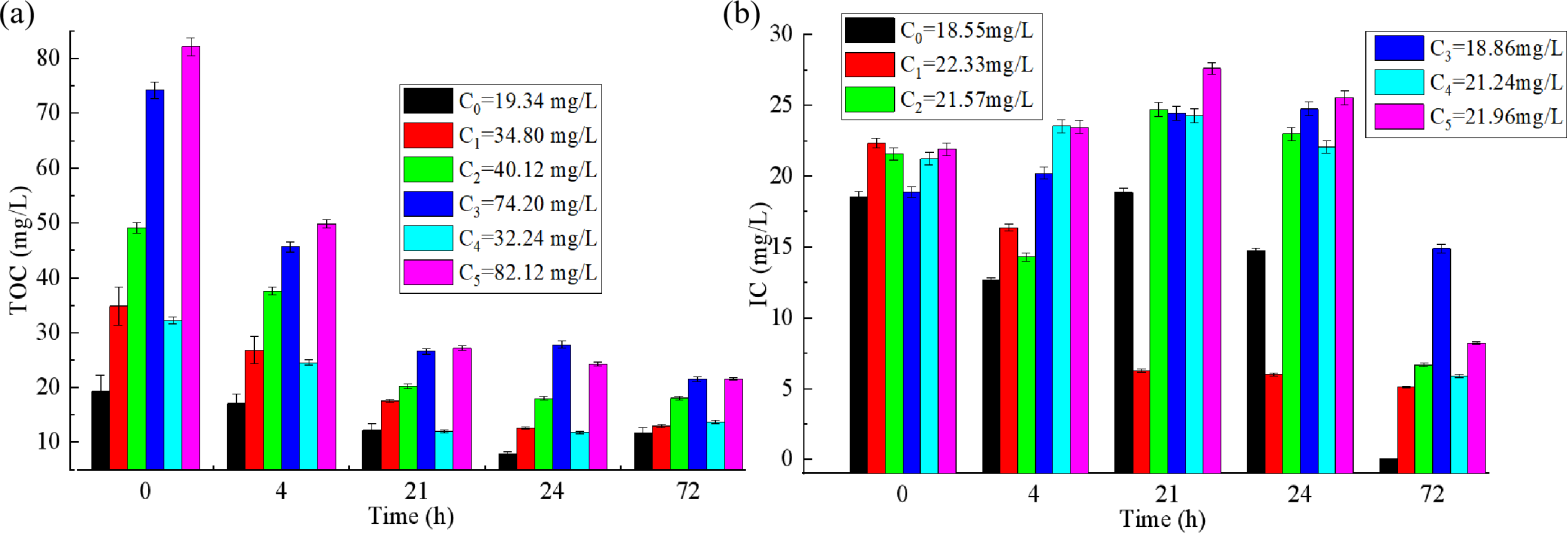
Temporal variations of TOC and IC concentrations within the PBR under different influent TOC concentrations. (**a**) TOC; (**b**) IC.

Figure 3 shows the temporal variations of TOC and IC concentrations in PBRs inoculated with 10 g (wet weight) CBC under different influent TOC levels. In the absence of external CO_2_ supplementation, microbial consortia mainly rely on organic pollutants as carbon sources. As shown in Fig. 3a, among all the tested influent TOC levels, the effluent TOC concentration tended to stabilize after 21 h (i.e., HRT = 21 h). The influent TOC concentrations were 19.34, 34.8, 40.12, 74.20, and 82.12 mg/L. Therefore, the corresponding removal rates were 36.66%, 49.63%, 58.88%, 64.18%, and 66.89%, respectively. These results compare favorably with previous work: for instance, Papadopoulos et al. (13) reported 70%–83% dissolved COD removal within nine days using a CBC dominated by *Leptolyngbya* sp. to treat brewery wastewater. Notably, higher initial TOC concentrations corresponded to higher removal rates (Fig. 3a), possibly due to enhanced bacterial activity under elevated organic loading, which accelerated degradation. Concurrently, in PBRs with higher organic input, IC levels did not decline but instead increased (Fig. 3b), indicating active heterotrophic respiration—a process that releases CO_2_ during organic matter decomposition. After 21 h, as organic substrate diminished, bacterial respiration weakened, while photosynthetic carbon uptake continued under light exposure, leading to a gradual decline in IC, as evident in Fig. 3b. This dynamic reflects a symbiotic interaction within the PBR: oxygen produced by algal/cyanobacterial photosynthesis supports bacterial oxidation of organics, while CO_2_ released from bacterial respiration is assimilated by photosynthetic microorganisms (14).

**Fig 4:**
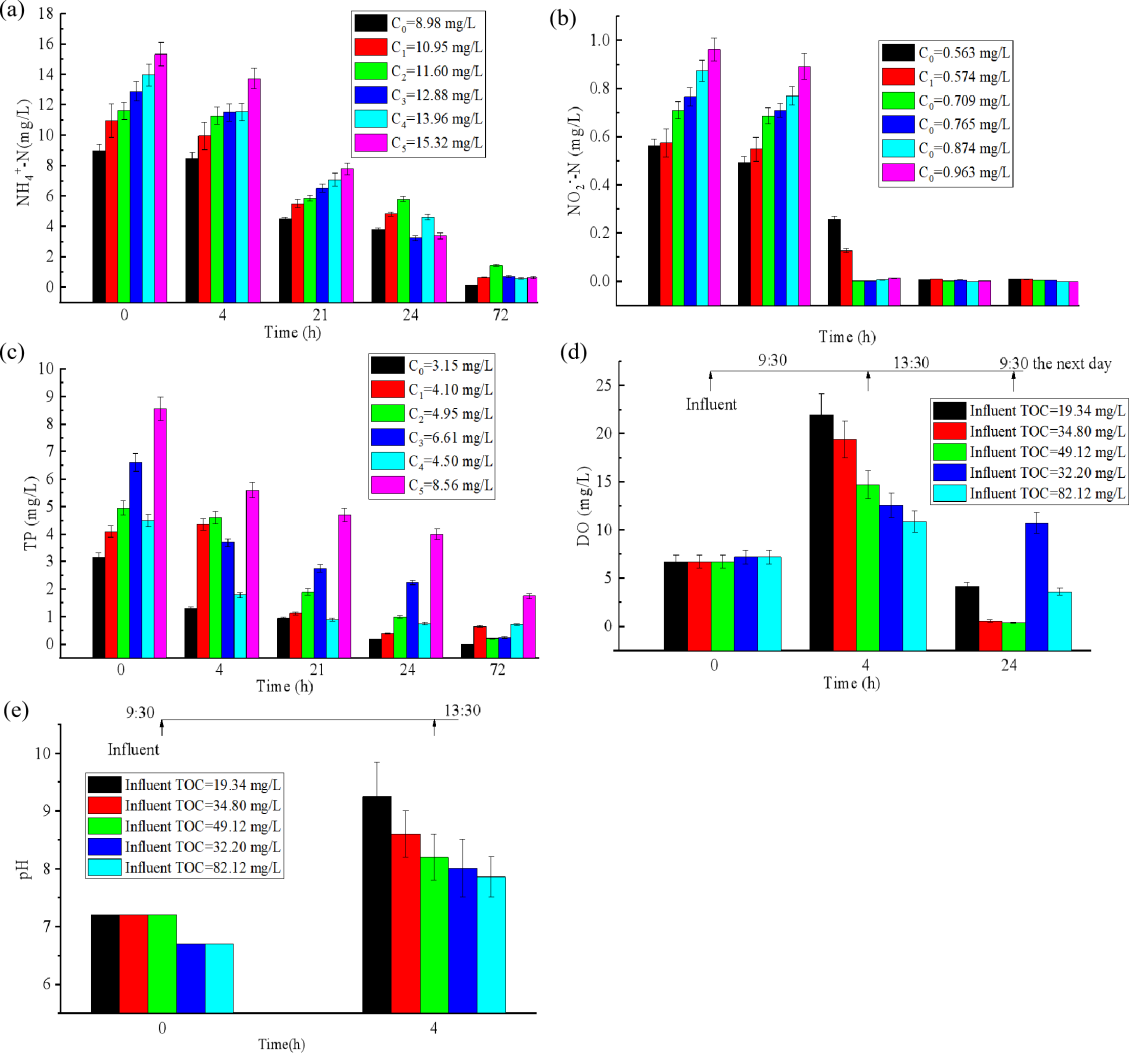
Temporal variations of water qualities in the PBRs under different influent pollutant concentrations. (**a**) NH_4_^+^-N; (**b**) NO_2_⁻-N; (**c**) TP;（**d**) DO concentrations; (**e**) pH value.

The temporal variations of NH_4_^+^-N, TP, and NO_2_⁻-N concentrations in the PBRs inoculated with 10 g (wet weight) CBC under different influent pollutant concentrations are shown in Fig. 4. As shown in Fig. 4a, the NH_4_^+^-N concentration in each PBR continuously decreased, with the removal rate of NH_4_^+^-N initially decreasing rapidly and then more gradually. After 21 h (i.e. HRT = 21 h), the NH_4_^+^-N removal rate in each PBR had reached approximately 50%. After 72 h (HRT = 72 h), the NH_4_^+^-N removal rates in the PBRs with initial NH_4_^+^-N concentrations of 8.98, 10.95, 11.60, 12.88, 13.96, and 15.32 mg/L were 100%, 94.02%, 87.60%, 94.39%, 95.84%, and 95.79%, respectively, with effluent NH_4_^+^-N concentrations all below 2 mg/L. This finding is consistent with the conclusions of Liu et al. (15). The preferential uptake of NH_4_^+^-N by algae and the nitrification of bacteria under high DO conditions are plausible explanations for the high NH_4_^+^-N removal rates (16).

As shown in Fig. 4b, after 21 h of treatment (HRT = 21 h), the NO_2_⁻-N removal rate in the PBRs with initial NO_2_⁻-N concentrations of 0.56, 0.57, 0.71, 0.76, 0.87, and 0.96 mg/L all reached 100%, with no new NO_2_⁻-N generated during the subsequent 3 days of operation. This is because, under light conditions, algae produce oxygen through photosynthesis, significantly increasing the DO in the PBR, which provides a suitable metabolic environment for microorganisms and accelerates the conversion of NO_2_⁻-N to NO_3_⁻-N. Additionally, the algae in the PBR can directly utilize NO_2_⁻-N as a nitrogen source (17), thereby collectively reducing the NO_2_⁻-N concentration in the wastewater.

As shown in Fig. 4c, phosphorus removal by the algal-bacterial consortium generally exhibited an initial rapid phase, followed by a slower uptake rate. After 24 h (HRT = 24 h), PBRs with influent TP concentrations below 5 mg/L achieved effluent TP levels around 1 mg/L, corresponding to removal efficiencies of approximately 80%–90%. In contrast, systems with influent TP above 6 mg/L maintained effluent TP above 2 mg/L, with removal rates ranging from 50% to 70%. After 72 h (HRT = 72 h), the TP removal efficiencies for initial concentrations of 3.15, 4.10, 4.50, 4.95, 6.61, and 8.56 mg/L reached 100%, 84.35%, 100%, 100%, 94.35%, and 79.63%respectively. These results demonstrate that the luxury uptake capacity of algae (18) can support high P removal. Notably, in the PBR with an influent TP of 8.56 mg/L, the effluent TP concentration remained above 2 mg/L even after 72 h. Although algae are effective at P assimilation (19), their uptake is often constrained by the N:P ratio (20). When the N:P ratio falls below 10:1, N becomes limiting, while P is in relative excess. In this experiment, N concentration was already low after 72 h due to prior microbial uptake, likely creating N-limited conditions that restricted further P removal by algae. Consequently, competition between bacteria and algae for available N may explain the residual P observed in the effluent.

The variations in DO and pH within the PBRs under different influent TOC concentrations are presented in Fig. 4d and e. As shown in Fig. 4d, the extent of DO increase was influenced by the influent TOC level: lower TOC concentrations resulted in a more pronounced rise in DO. This trend can be attributed to the reduced oxygen demand for heterotrophic bacterial metabolism under lower organic loading, allowing a greater proportion of the oxygen generated via algal photosynthesis to remain in the system. Conversely, higher TOC concentrations led to greater oxygen consumption by heterotrophic bacteria, thus limiting the net increase in DO. Despite these differences, DO in all PBRs reached saturation around 13:30 (midday), demonstrating that even without external aeration, algal photosynthesis can maintain high DO levels in the reactor. Subsequently, after a night passed, DO decreased in each PBR due to the absence of sunlight, which halted photosynthetic oxygen production. These observations confirm a diurnal fluctuation in DO, with peak values occurring around noon and lower levels sustained during the night and early morning.

As illustrated in Fig. 4e, the extent of pH increase varied with influent TOC concentration. Lower TOC levels led to a more pronounced rise in pH, a trend that closely paralleled the observed DO dynamics in the PBRs. For instance, when the influent TOC was 82.12 mg/L, pH increased from 6.7 to 7.7 after 4 h of sunlight exposure (irradiance: 55,500–69,900 Lux). In contrast, under the same light conditions, the pH in the PBR with an influent TOC of 19.34 mg/L rose from 7.2 to approximately 9.2. This pattern can be explained by the interplay between heterotrophic bacterial activity and algal photosynthesis. At lower TOC concentrations, less IC is generated through bacterial respiration, creating an IC deficit that limits algal carbon assimilation. Consequently, alkalinity decreases and pH rises more sharply as CO_₂_ is consumed. Conversely, higher TOC levels stimulate greater bacterial IC production, which buffers the system and attenuates the pH increase.

### Performance of PBRs in treating actual MWW

**Fig 5:**
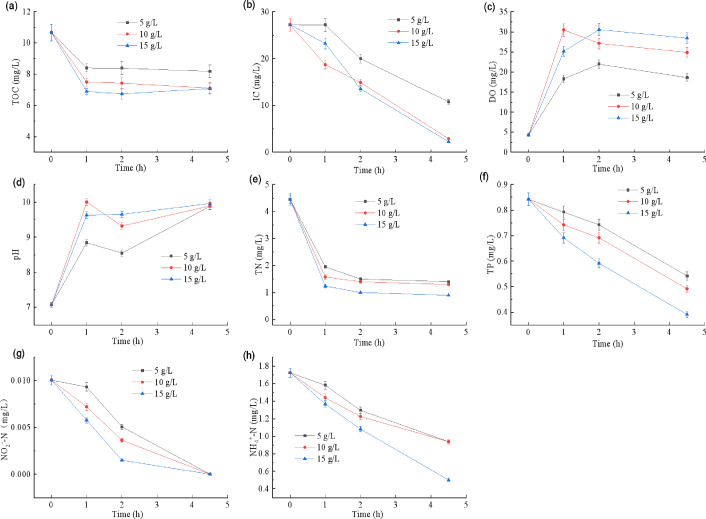
Water quality changes with time in PBRs inoculated with different qualities of CBC for treating actual MWW. The PBRs were operated under natural sunlight from 9:30 to 14:00, with continuous stirring to maintain mixing of the CBC and wastewater. (**a**) TOC; (**b**) IC; (**c**) DO; (**d**) pH; (**e**) TN; (**f**) TP; (**g**) NO₂⁻-N; (**h**) NH₄^+^-N.

The treatment performance of PBRs inoculated with different quantities of the CBC on actual shrimp WMM is presented in Fig. 5. Compared with the PBR inoculated with 5 g of wet CBC (5_X), the reactor inoculated with 15 g (15_X) exhibited faster removal rates for TC, TOC, IC, TN, TP, NH₄^+^-N, and NO₂⁻-N. After 1 h, TOC in 15_X decreased from 10.68 to 6.89 mg/L, TN from 4.45 to 1.24 mg/L, TP from 0.84 to 0.69 mg/L, NH₄^+^-N from 1.72 to 1.37 mg/L, and NO₂⁻-N from 0.01 to approximately 0.006 mg/L. And after 4.5 h, the TOC removal efficiencies for 15_X, 10_X, and 5_X were 33.72%, 33.44%, and 23.37%, respectively, with effluent TOC concentrations ranging between 7 and 8  mg/L. Corresponding TN removal rates reached 79.78%, 70.79%, and 68.54%, with effluent TN levels of 0.9–1.5  mg/L. TP removal efficiencies were 53.56%, 41.81%, and 35.71%, yielding effluent TP values of 0.39–0.54  mg/L. For NH₄^+^-N, the removal rates were 45.6%, 46.6%, and 71%, resulting in effluent NH₄^+^-N concentrations of 0.50–0.94  mg/L. Notably, NO₂⁻-N was completely removed (to below detection) in all three PBRs from an initial concentration of 0.01 mg/L.

Simultaneously, as shown in Fig. 5, a higher inoculum mass of the CBC led to a more rapid increase in pH and DO within the PBRs. After 1 h, the pH in reactors 10_X and 15_X rose from approximately 7 to 9.5–10, while DO increased from 4.6 mg/L to 23–30 mg/L. In contrast, in PBR 5_X, pH increased only to about 8.7 and DO reached 17.5 mg/L over the same period. With extended time, however, the differences diminished; after 4.5 h, pH values in all PBRs converged near 9.7, and DO declined slightly due to reduced light intensity but remained positively correlated with algal biomass.

Overall, a larger algal inoculum accelerated pollutant removal. It is noteworthy, however, that the rapid consumption of IC coupled with the steep rise in pH and DO driven by intense algal photosynthesis may indicate carbon limitation and potential physiological stress on the consortium under high-density conditions.

### Growth of the CBC in PBRs

**Fig 6:**
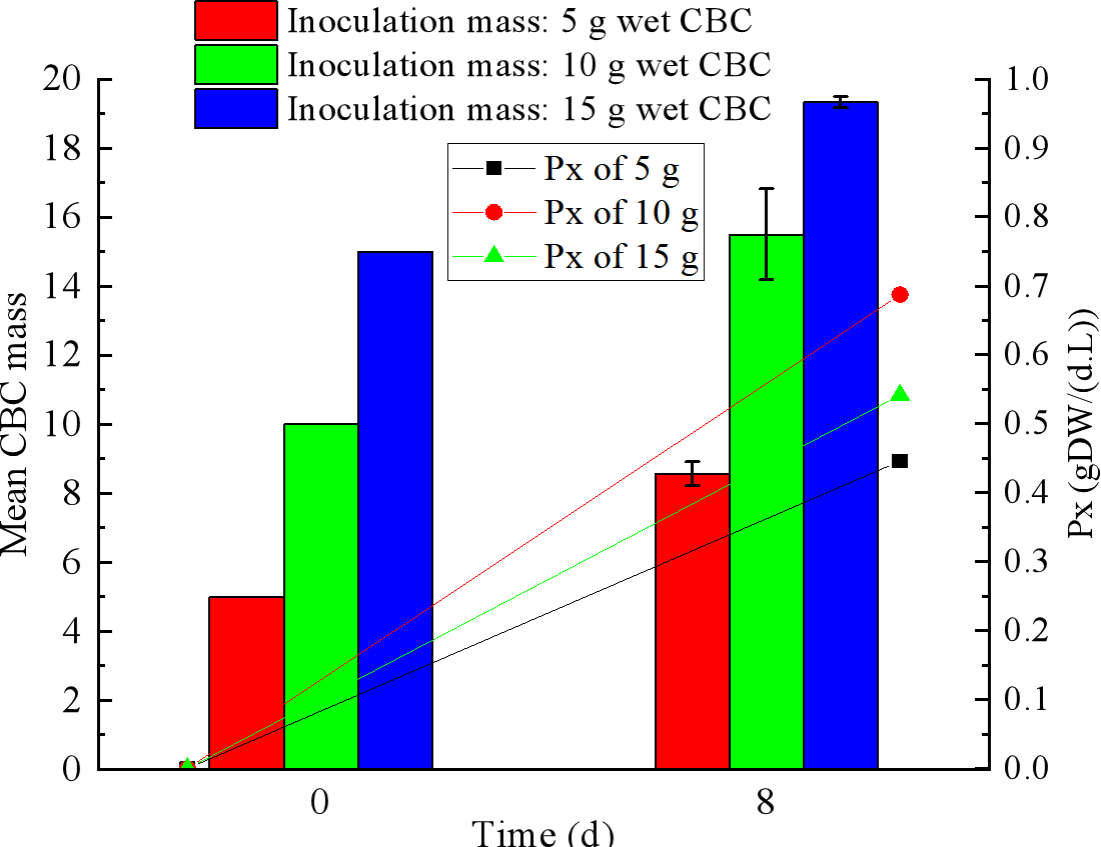
Growth of the CBC in PBRs for treating actual shrimp MWW.

The CBC was cultivated continuously in PBRs for treating actual MWW from shrimp pond for 8 days. The growth rate of CBC is shown in Fig. 6, and *P*_x_ was determined. The growth of CBC is mainly influenced by light, C, N, and P. The nutrients present in the actual shrimp MWW cause the CBC to increase in quality under light conditions.

### Analysis of microbial composition and relative abundance in the CBC for treating actual MWW

#### Bacterial diversity and composition

To investigate the dynamic changes of the population in CBC after treating MWW from shrimp pond, high-throughput sequencing was performed for samples from three PBRs with different inoculum qualities on day 9 of the experiment and indigenous CBC.

**Fig 7:**
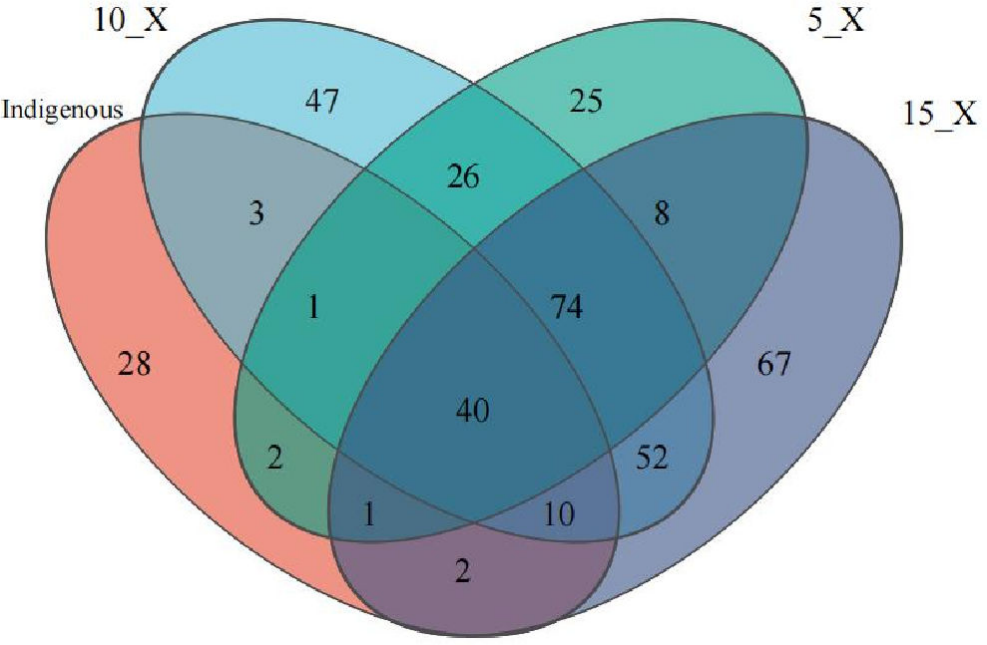
Venn diagram of species.

A Venn diagram was used to compare unique and shared bacterial genera among the indigenous community and the PBR systems (5_X, 10_X, and 15_X) (Fig. 7). Following cultivation with shrimp pond wastewater, the diversity and abundance of both bacterial and algal taxa increased substantially in all PBRs. Only three bacterial genera were shared between the indigenous community and the cultivated systems, whereas over a hundred new genera were detected after treatment. The 5_X, 10_X, and 15_X reactors contained 141, 200, and 194 bacterial genera, respectively. The 15_X system exhibited the highest number of unique species (67), compared with only 25 in 5_X. A total of 146 genera were shared between 15_X and 10_X, accounting for 75% and 73% of their respective total genera. All three reactors shared 96 bacterial genera, representing 68%, 48%, and 49% of the total genera in 5_X, 10_X, and 15_X, respectively. These results indicate that both inoculum characteristics and wastewater composition influenced microbial abundance and bacterial diversity within the PBRs.

**Fig 8:**
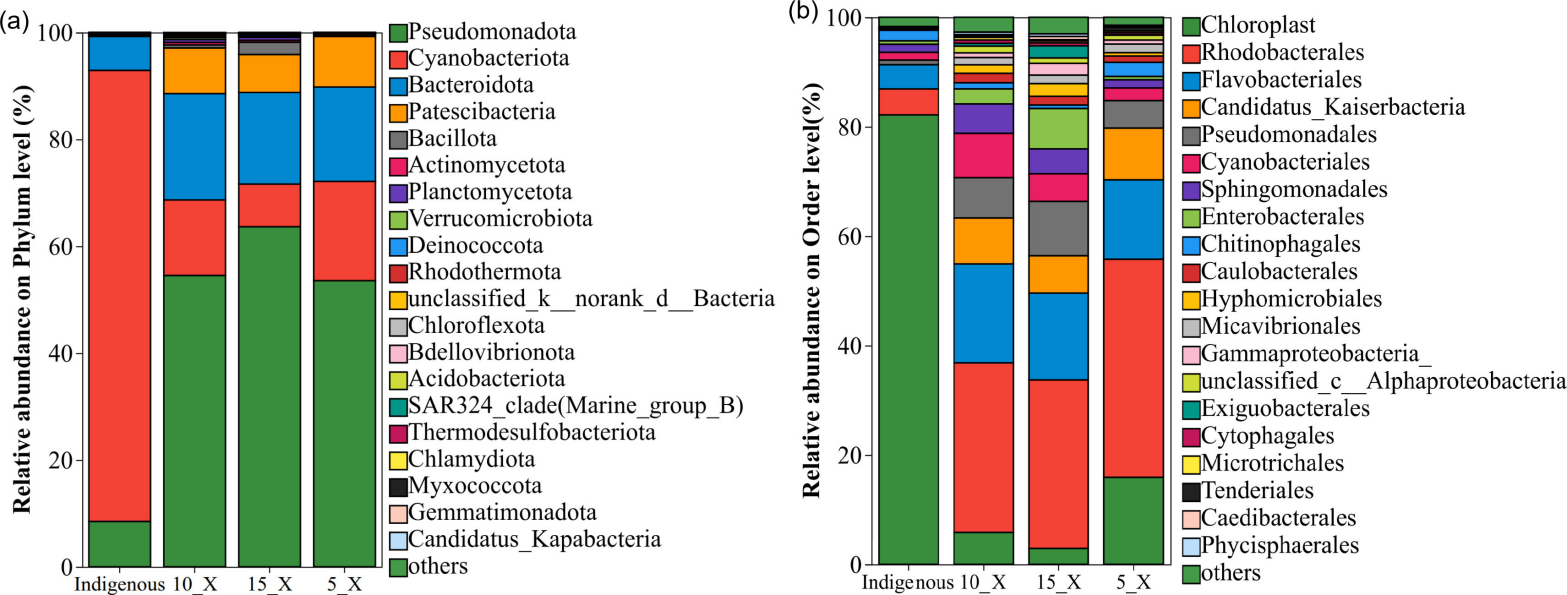
Relative abundance of bacteria in reactors 5_X, 10_X, and 15_X. (**a**) Phylum level; (**b**) order level.

Figure 8 illustrates the relative abundance of bacterial phyla in the indigenous community (CBC) and within the PBR systems (5_X, 10_X, and 15_X) during the treatment of shrimp MWW. Alpha diversity analysis further revealed that the relative abundances of Pseudomonadota, Bacteroidota, and Patescibacteria increased significantly over the cultivation period. In contrast, the phylum Cyanobacteriota exhibited a marked decline in relative abundance from an initial 84.4% to 18.58%, 14.03%, and 7.98% in 5_X, 10_X, and 15_X, respectively, with increasing inoculum quality and cultivation time. The increase in the abundance of other types of bacteria is the main reason for the decline in their relative abundance.

The predominant phyla across all three PBRs were Pseudomonadota, Bacteroidota, and Cyanobacteriota, which aligns with the findings reported by Rong et al. (21). Pseudomonadota represented the largest proportion, accounting for 53.58%, 54.66%, and 63.76% in 5_X, 10_X, and 15_X, respectively. Bacteroidota followed, with relative abundances of 17.54%, 19.75%, and 17.24% in the corresponding reactors. Zhou et al. (22) observed that algal growth suppressed Bacteroidota while promoting Pseudomonadota, a trend that was more pronounced in the 15_X reactor in the present study. This could be attributed to the greater availability of organic matter derived from enhanced algal photosynthesis under higher inoculum conditions, thereby fostering the proliferation of Pseudomonadota. Supporting this, Pseudomonadota are recognized as effective contributors to organic matter and nitrogen removal under high-salinity conditions (23), and they commonly dominate laboratory-scale wastewater treatment bioreactors, comprising approximately 40%–50% of the bacterial community (24). In microalgal symbiotic systems, Patescibacteria are dominant and are believed to play a key role in phycosphere nutrient cycling, with their considerable diversity, suggesting a rapid evolutionary rate within this group (25).

At the order level, the relative abundance of Chloroplast, the most abundant order within the Cyanobacteria phylum (26), declined substantially from 82.15% in the indigenous CBC to 2.91% in 15_X, 5.84% in 10_X, and 15.91% in 5_X. In contrast, Rhodobacterales increased markedly from 4.75% in the indigenous CBC to 30.80% in 15_X, 30.96% in 10_X, and 39.87% in 5_X. Flavobacteriales also rose significantly, from 4.36% in the indigenous CBC to 15.87% in 15_X, 18.08% in 10_X, and 14.50% in 5_X, a third notable increase was observed for Candidatus_Kaiserbacteria, which increased from undetectable levels to 6.83%–9.47%. Additionally, Pseudomonadales, Cyanobacteriales, and Enterobacterales exhibited upward trends in relative abundance.

Rhodobacterales, belonging to the class Alphaproteobacteria, are metabolically versatile microorganisms capable of both photoheterotrophic and chemoheterotrophic growth. They demonstrate adaptability to aerobic, anaerobic, and microaerobic conditions and play a key role in global carbon and sulfur cycling, including the degradation of dimethylsulfoniopropionate (27). Flavobacteriales, within the phylum Bacteroidota, constitute an important link in the “planktonic bacteria-phytoplankton” degradation cycle, capable of degrading polysaccharides from diatom and dinoflagellate cell walls, releasing dissolved organic carbon, and thereby facilitating energy recycling in microbial systems (28). Candidatus_Kaiserbacteria represents a widespread yet uncultured lineage of ultra-small bacteria that may participate in carbon-nitrogen cycling through denitrification, methanogenesis, or symbiotic interactions, potentially fine-tuning pollutant degradation or plant-microbe dynamics in certain environments (29).

Collectively, these shifts indicate that during wastewater treatment, the originally dominant taxa in the indigenous community decreased in abundance, while taxa better adapted to the wastewater conditions became increasingly prevalent.

**Fig 9:**
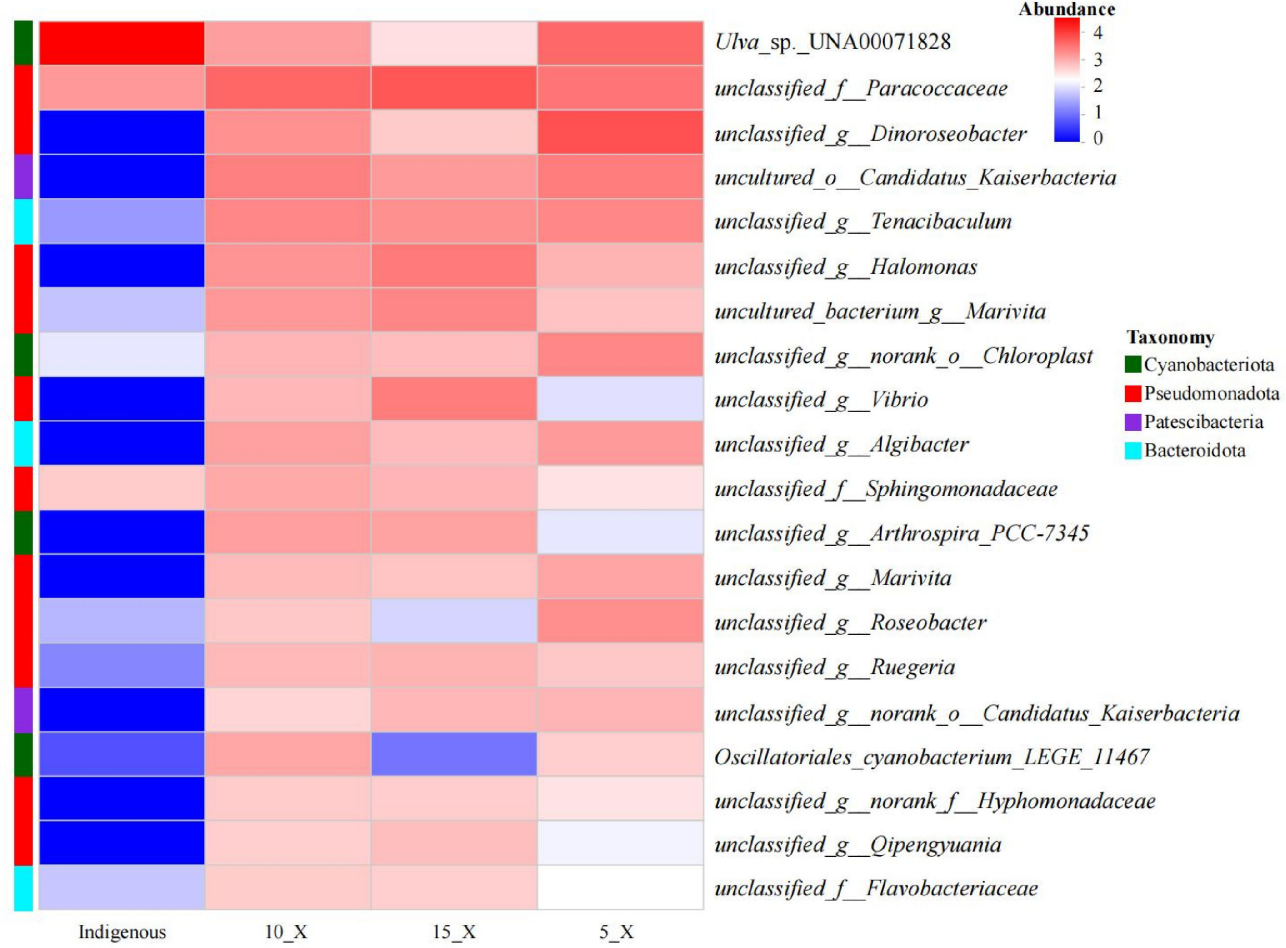
Community heatmap on species level. The x-axis represents sample names, and the y-axis represents species names. The color boxes represent the abundance of each species.

The heatmap (Fig. 9) intuitively illustrates the distribution of the top 20 dominant bacterial species across different samples. Notably, the unclassified family Paracoccaceae has replaced the macroalga *Ulva* sp. UNA00071828 as the dominant taxon within the three PBRs treating wastewater. Paracoccaceae belongs to the phylum Pseudomonadota, class Alphaproteobacteria, and order Rhodobacterales, with members exhibiting extensive metabolic capabilities and the ability to utilize a wide range of carbon and nitrogen sources. Additionally, taxa belonging to the order Candidatus_Kaiserbacteria (phylum Patescibacteria) showed a significant increase in abundance in the PBRs, despite being present at very low levels in the original CBC. These bacteria typically grow as epibionts on the surface of larger host microbial cells (25).

The genus *Marivita* also became notably enriched in the PBRs. Belonging to the family Rhodobacteraceae, *Marivita* comprises strictly aerobic bacteria (30) that typically contain genes for aerobic anoxygenic photosynthesis (AAP) and produce bacteriochlorophyll a. These organisms can utilize diverse carbon and nitrogen sources (31) and are widely distributed in marine environments, especially in algal-associated microenvironments. They have been found to interact dynamically with algae through cross-kingdom algal–bacterial interactions (ABI), likely playing important roles in algal growth and metabolism (31) . The fourth dominant genus, *Tenacibaculum* (family Flavobacteriaceae), is known to cause various diseases in aquaculture (32) and may therefore originate from the aquaculture wastewater itself. The fifth dominant taxon in the PBRs was *Arthrospira* PCC-7345 (phylum Cyanobacteria, order Oscillatoriales, family Microcoleaceae), a filamentous cyanobacterium identified as *Arthrospira platensis* strain PCC-7345. Its relative abundance increased from 0.07% in the indigenous CBC to 3.24% (15_X), 3.25% (10_X), and 2.43% (5_X). Filaments of this species are typically spiral or linear, approximately 10–15 μm in width, and consist of multicellular chains. Capable of oxygenic photosynthesis, it is rich in proteins, polysaccharides, and pigments such as chlorophyll and carotenoids. The strain grows optimally at 20°C and tolerates high-salinity conditions (e.g., 150 g/L NaCl) (33).

The genus *Dinoroseobacter* (family Rhodobacteraceae) also increased in relative abundance from 0.08% in the indigenous CBC to 2.73% (15_X), 3.22% (10_X), and 3.77% (5_X). Possessing AAP genes and producing bacteriochlorophyll a, *Dinoroseobacter* can perform photosynthesis under light and thrives in seawater environments. Studies of marine planktonic microenvironments indicate that *Dinoroseobacter* dynamically interacts with algae through ABI, potentially influencing algal growth and metabolism (34). *Roseobacter* (family Rhodobacteraceae, phylum Proteobacteria), which also carries bacteriochlorophyll a and performs AAP, increased from 1.65% in the indigenous CBC to 1.91% (15_X), 2.74% (10_X), and 3.24% (5_X). Members of *Roseobacter* can metabolize organic and inorganic sulfur and are capable of oxidizing carbon monoxide, a trait that may represent an important response to photolysis of dissolved organic matter (35). The genus *Algibacter* (phylum Bacteroidota, order Flavobacteriales, family Flavobacteriaceae) increased from 0.08% in the indigenous CBC to 2.88% (15_X), 3.08% (10_X), and 3.14% (5_X). These aerobic or facultatively anaerobic heterotrophs can degrade a variety of organic substrates, including algal polysaccharides, and are commonly found in marine algal microenvironments. They possess a rich repertoire of carbohydrate-active enzymes, enabling efficient degradation of algal polysaccharides (36).

### Conclusion

An indigenous CBC was inoculated into PBRs to treat both synthetic and actual MWW. The introduction of CBC significantly increased the DO concentration in the MWW within the PBRs. In experiments with synthetic MWW, under varying initial NH₄^+^-N concentrations (8.98–15.32 mg/L), the NH₄^+^-N removal efficiency by CBC-inoculated PBRs was consistently high (90%–100%) at a HRT of 3 days. When the initial NO₂⁻-N concentration ranged from 0.56 to 0.96 mg/L, complete removal (100%) of NO₂⁻-N was achieved at an HRT of 21 h. TP removal efficiency reached approximately 80%–90% after 24 h of HRT. For actual shrimp MWW treated at an HRT of 4.5 h, PBRs inoculated with 15, 10, and 5 g/L CBC (wet weight) achieved TOC removals of 33.72%, 33.44%, and 23.37%; TN removals of 79.78%, 70.79%, and 68.54%; and TP removals of 53.56%, 41.81%, and 35.71%, respectively. Microbial community analysis revealed that the dominant phyla in PBRs treating actual shrimp pond wastewater were Pseudomonadota, Bacteroidota, and Cyanobacteriota. Family Paracoccaceae replaced the macroalga *Ulva* sp. UNA00071828 as the dominant taxon across all PBRs. Additionally, the genus *Marivita* (family Rhodobacteraceae) and order Candidatus_Kaiserbacteria were also notably enriched in the PBRs treating MWW.

## Data Availability

Data can be obtained from the following link: https://www.ncbi.nlm.nih.gov/sra/PRJNA1435590.
